# A NAC transcription factor OsNAC3 positively regulates ABA response and salt tolerance in rice

**DOI:** 10.1186/s12870-021-03333-7

**Published:** 2021-11-20

**Authors:** Xiang Zhang, Yan Long, Xingxiang Chen, Baolei Zhang, Yafeng Xin, Longying Li, Shuling Cao, Fuhang Liu, Zhigang Wang, Hao Huang, Degui Zhou, Jixing Xia

**Affiliations:** 1 State Key Laboratory for Conservation and Utilization of Subtropical Agro-bioresources, College of Life Science and Technology, Guangxi University, Nanning, 530004 China; 2 Guangdong Key Laboratory of New Technology in Rice Breeding, Rice Research Institute, Guangdong Academy of Agricultural Sciences, Guangzhou, 510640 China

**Keywords:** Salinity, Abscisic acid, Salt tolerance, Transcription factor, Rice

## Abstract

**Background:**

NAC (NAM, ATAF and CUC) transcription factors (TFs) play vital roles in plant development and abiotic stress tolerance. Salt stress is one of the most limiting factors for rice growth and production. However, the mechanism underlying salt tolerance in rice is still poorly understood.

**Results:**

In this study, we functionally characterized a rice NAC TF OsNAC3 for its involvement in ABA response and salt tolerance. ABA and NaCl treatment induced *OsNAC3* expression in roots. Immunostaining showed that OsNAC3 was localized in all root cells. *OsNAC3* knockout decreased rice plants’ sensitivity to ABA but increased salt stress sensitivity, while *OsNAC3* overexpression showed an opposite effect. Loss of *OsNAC3* also induced Na^+^ accumulation in the shoots. Furthermore, qRT-PCR and transcriptomic analysis were performed to identify the key OsNAC3 regulated genes related to ABA response and salt tolerance, such as *OsHKT1;4, OsHKT1;5, OsLEA3–1, OsPM-1, OsPP2C68,* and *OsRAB-21.*

**Conclusions:**

This study shows that rice OsNAC3 is an important regulatory factor in ABA signal response and salt tolerance.

**Supplementary Information:**

The online version contains supplementary material available at 10.1186/s12870-021-03333-7.

## Background

Rice (*Oryza sativa* L.) is the major staple cereal food for over one-third of the world’s population. Unfortunately, rice yield and grain quality are often affected by adverse environmental factors such as salinity, drought, or inappropriate temperature [1–3]. Especially, high salinity severely impacts rice growth and harvest [4]. During evolution, plants developed numerous sophisticated but effective strategies to counter such adverse conditions. For instance, plant transcription factors (TFs) can activate a series of stress-related genes to synthesize diverse functional proteins that enable plant survival. TFs WRKY [5], bZIP [6], MYB [7], and NAC [8] have been well characterized for their important roles in plant stress or defense responses.

Among the stress-related TFs, the NAC (NAM, ATAF, CUC1/2) family of TFs has acquired great attention for their role in abiotic stress resistance [9]. So far, 151 rice and 117 *Arabidopsis* NAC proteins have been identified [10]. Normally, NAC proteins consist of a conserved DNA-binding domain (NAC domain) in the N-terminal region; on the contrary, the transcriptional regulating C-terminal domain usually varies in both amino acid composition and biological function [11]. NAM from *petunia* [12] and CUC2 from *Arabidopsis* [13] are one of the first characterized NAC genes, which participate in the shoot apical meristem development. NAC genes have received significant attention for their regulatory role in plant organ development and stress resistance [11]. For example, AtNAC1 regulates lateral root development in *Arabidopsis* via auxin signal transduction [14]. OsNAC2 gets significantly upregulated during leaf senescence and dramatically accelerates the process in rice [15]. Downregulation of *ONAC122* or *ONAC131* enhances rice susceptibility to *Magnaporthe grisea* [16]. ABA, salt, drought and cold stresses induced *SNAC2* conferring drought and salt tolerance in transgenic rice [17]. The *RD26* gene is an important regulator of ABA-dependent stress response in *Arabidopsis* [18].

Apart from stress-related TFs that directly regulate the expression of abiotic stresses related genes, phytohormones such as ABA also play a critical role in plant abiotic resistance [19]. Stress conditions upregulate the ABA-biosynthesis genes to increase the ABA content [20]. Notably, under abiotic stresses, NAC genes can also regulate the ABA signal transduction pathway. For example, in rice, over-expression of *OsNAC2* confer to drought and salt resistance and ABA biosynthesis gene *OsNCED3* is upregulated [21]. OsNAP expression profile in rice showed that ABA and abiotic stresses such as high salinity, drought, and low temperature significantly induced the expression level of *OsNAP* [22]. Transgenic plants overexpressing OsNAC52 showed high sensitivity to ABA [23].

Previously, OsNAC3 was suggested to be involved in rice stress responses [24]. However, the exact functions of OsNAC3 were not investigated. Here, we examined the expression profile of *OsNAC3* and physiological phenotypes using its knockout and overexpressing transgenic rice lines. High throughput RNA-seq assay was performed to find the downstream target genes of *OsNAC3*. We show that OsNAC3 positively regulates the ABA pathway and salt tolerance in rice via the regulation of *OsHKT1;4, OsHKT1;5, OsLEA3–1, OsPM-1, OsPP2C68,* and *OsRAB-21.* These findings can help the development of better salt-tolerant crops.

## Results

### Sequence analysis of *OsNAC3*

The full-length open reading frame of *OsNAC3* (Os07g0225300) was cloned based on the Rice Annotation Project Database (RAP-DB, http://rapdb.dna.affrc.go.jp/). We used the OsNAC3 amino acid sequence as a query to search for its homologs in rice and *Arabidopsis*, and 17 homologs with more than 75% identity were obtained. These proteins, together with OsNAC3 and OsNAC45, were used to construct the phylogenetic tree. Phylogenetic analysis showed that OsNAC3 is closely related to the NAC family of TFs (SNAC1 and OsNAC4) in rice (Additional file 1 Fig. S1 A). It contains a single exon encoding the 277 amino acids protein with a highly conserved N-terminal NAC domain, which can be divided into five subdomains(A-E)(Additional file 1 Fig. S1 B). Additionally, many stress-related cis-acting elements were found in the promoter region (2 kb upstream from the start codon) of the *OsNAC3* gene, including nine MYB binding sites, six MYC binding sites, three W-boxes, two ABRE(ABA-responsive element)sites, and three As-1 sites (Additional file 1 Fig. S1 C).

### Expression pattern of *OsNAC3*

**Fig. 1:**
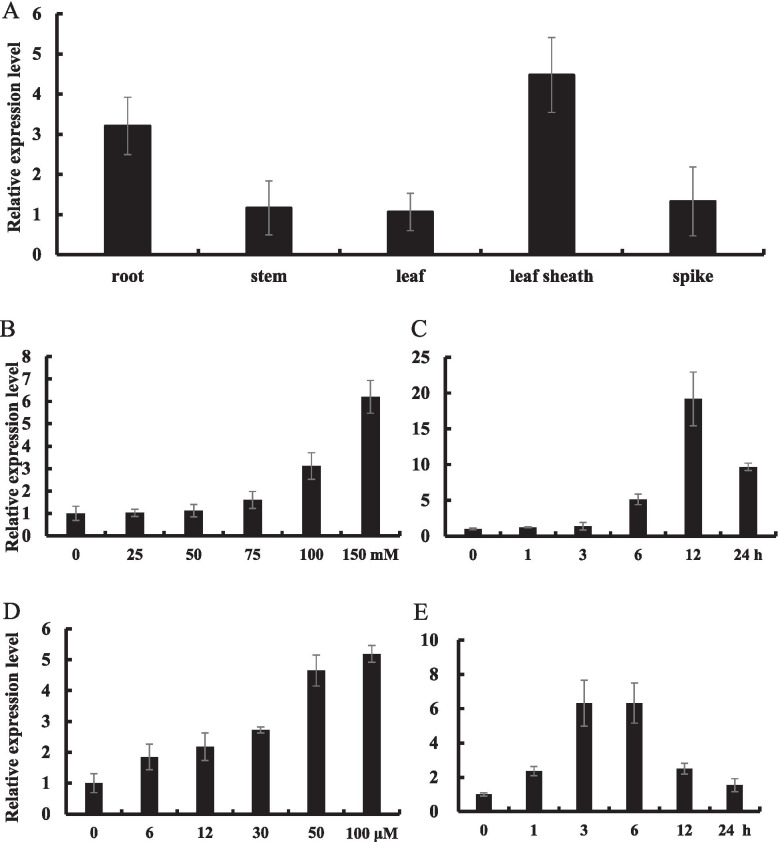
Expression profile of *OsNAC3*. (**A**) *OsNAC3* expression in the root, stem, leaf, leaf sheath, and spike of heading stage rice. (**B**) Dose-dependent expression level of *OsNAC3* in rice roots under salt treatment. 5-day-old rice seedlings were exposed to different concentrations of NaCl (0, 25, 50, 75, 100, and 150 mM) for 12 h. (**C**) Time-course expression level of *OsNAC3* in rice roots under 100 mM NaCl treatment. 5-day-old rice seedlings were exposed to 100 mM NaCl for different times (0, 1, 3, 6, 12, and 24 h). (**D**) Dose-dependent expression level of *OsNAC3* in rice roots under ABA treatment. 5-day-old rice seedlings were exposed to different concentrations of ABA (0, 6, 12, 30, 50, and 100 μM) for 12 h. (**E**) Time-course expression level of *OsNAC3* in rice roots under 50 μM ABA treatment. 5-day-old rice seedlings were exposed to 50 μM ABA at different times (0, 1, 3, 6, 12, and 24 h). *Histone H3* was used as an internal standard. Data represent means ± SD (*n* = 3)

The expression level of *OsNAC3* in root, stem, leaf, leaf sheath, and spike were investigated with qRT-PCR. The results showed that the highest expression of *OsNAC3* was in leaf sheath, and the lowest expression was in stem and leaf (Fig. 1A). To investigate the possible role of *OsNAC3* in abiotic stress resistance, we examined its expression in rice roots after salt (NaCl) treatment in a time and dose gradient manner. Notably, *OsNAC3* expression was the highest at 12 h and showed a positive correlation with the concentration of NaCl (Fig. 1B, C). Considering ABA’s role in plant abiotic stress response, we investigated *OsNAC3* expression after rice treatment with exogenous ABA. We found that *OsNAC3* expression was the highest during 3–6 h treatment of 100 μM ABA, and showed a positive correlation with ABA concentration (Fig. 1D, E).

### Subcellular and cellular localization of OsNAC3

**Fig. 2:**
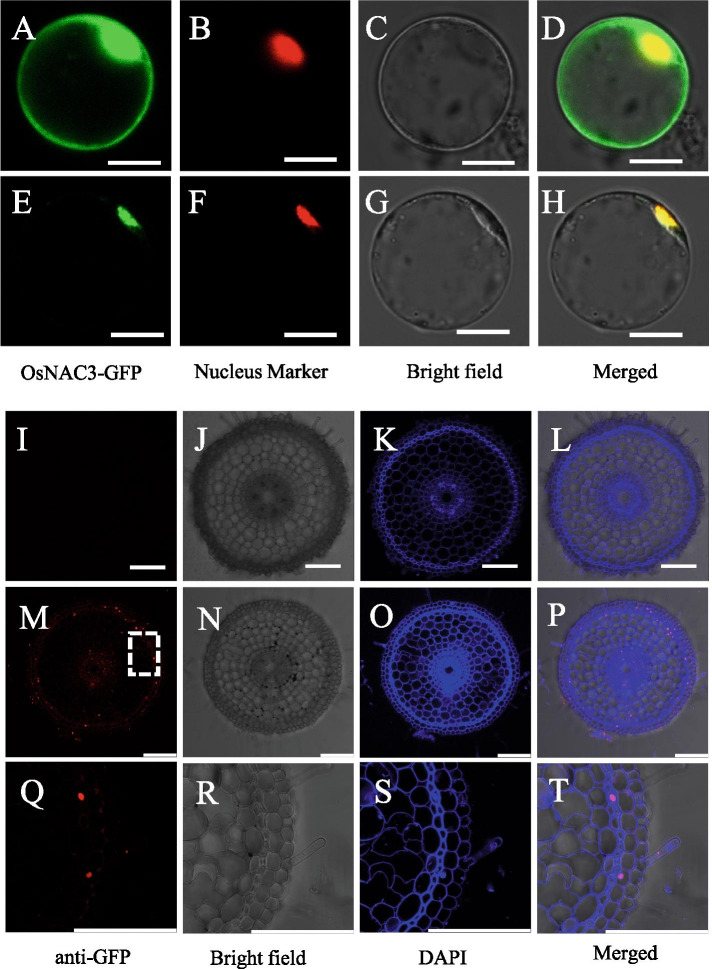
Subcellular and cellular localization of OsNAC3. (**A-H**) Rice protoplast co-expressing *GFP-OsNAC3* or *GFP* with *OsGhd7* (nuclear marker) under the control of CaMV35S promoter. The protoplasts were isolated from the leaf sheaths of 14-day-old rice seedlings (9311, *Oryza sativa* L. ssp.*indica*). Scale bar = 10 μm. (**I-T**) Immunostaining of the roots of 7-day-old Nipponbare rice (upper panels) and Pro*OsNAC3-OsNAC3-GFP* transgenic plants (middle panels) with anti-GFP antibodies. High-magnification images (Q-T) of the dotted part of the middle panels (M-P). The red and blue colors are the signal of anti-GFP antibody and DAPI staining of cell walls and nuclei, respectively. Scale bar = 20 μm

To investigate the subcellular localization of OsNAC3, we fused its coding region with green fluorescence protein (GFP) and introduced it into rice protoplasts along with nuclear marker *OsGhd7-RFP*. The green fluorescence of the control vector (35S: GFP) was observed in the whole cell, while the green signal of OsNAC3 nicely overlapped with the red fluorescence of OsGhd7-RFP (Fig. 2A-H), indicating nuclear localization of OsNAC3.

To further investigate the cellular localization of OsNAC3 in rice roots, Pro*OsNAC3-OsNAC3*-*GFP* transgenic rice was constructed. Immunostaining assay was performed with GFP and DAPI was used as a nuclear stain. Antibodies against GFP (red signal) were detected in all the cell layers of transgenic roots, while it nicely overlapped with DAPI stain (blue signal) in the nucleus (Fig. 2M-T). This confirmed the nuclear localization of OsNAC3 in root cells. Meanwhile, the red signal was absent in wild-type roots indicating the antibody specificity (Fig. 2I-L).

### Transcriptional activation analysis of OsNAC3

To examine the transcriptional activation activity of OsNAC3, we fused its coding region with the GAL4 DNA binding domain in the pGBK-*OsNAC3* vector, which was then transformed into yeast strain AH109. If OsNAC3 has transcriptional activation activity, GAL4-OsNAC3 binding to the GAL1 upstream sequence will activate the HIS3 reporter gene and restore the growth of the host yeast strain on a histidine-deficient plate. The results of the assay showed that the yeast cells carrying pGBK-*OsNAC3* and positive control cells grew normally on the SD/Trp- and SD/Trp−/His−/Ade- plates, while the negative control (pGBK-T7) only grew on the SD/Trp-, but not SD/Trp−/His−/Ade- plates (Additional file 1 Fig. S2). These results indicated that full-length OsNAC3 has self-activation activities in yeast.

### ABA affect the growth of *OsNAC3*-transgenic rice

Given that exogenous ABA induces the expression of *OsNAC3*, we speculate that exogenous ABA treatment may affect the growth of *OsNAC3*-transgenic rice. To test this hypothesis, we established an *OsNAC3*-knockout line (MT-1, MT-2) with CRISPR/Cas9 method and a maize ubiquitin promoter-driven *OsNAC3*-overexpression line (OE-1, OE-2) (more details in Additional file 1 Fig. S3, S4).

**Fig. 3:**
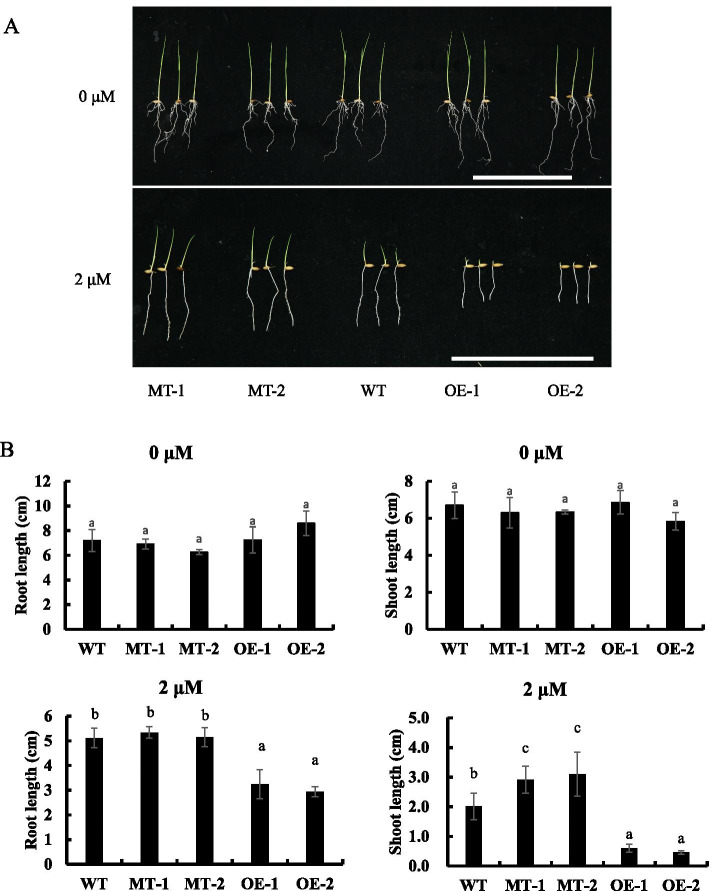
The growth of MT and OE plants under ABA treatment. (**A**) Growth performance of WT, MT, and OE seedlings under normal conditions for 5 days (upper panels). Growth performance of WT, MT, and OE seedlings under ABA treatment for 5 days (lower panels). Scale bar = 10 cm. (**B**) Shoot and root length of WT, MT, and OE seedlings under 0 or 2 μM ABA treatment for 5 days. All seeds were germinated on 1/2-strength MS medium without ABA for 2 days before the ABA treatment. Data represent means ± SD (*n* = 10). Different letters indicate significant differences (Tukey test, *p* < 0.05)

The seeds of *OsNAC3*-knockout, *OsNAC3*-overexpression, and wild-type rice were germinated in 1/2 MS medium for 2 days. Next, seedlings of similar size were transferred to a new 1/2 MS medium containing 0 or 2 μM ABA; after 5 days of cultivation, seedlings were photographed, and the lengths of roots and shoots were recorded. Results showed that in the absence of ABA, the growth of WT and *OsNAC3*-transgenic lines showed no obvious difference, indicating no effect of change in *OsNAC3* expression level under normal conditions. However, in the presence of exogenous ABA, the shoot and root growth of OE lines were severely inhibited compared with WT lines. The shoot length of MT lines was longer than WT lines, while root length was similar between WT and MT lines (Fig. 3A, B). These results demonstrated that overexpression of *OsNAC3* increased the ABA sensitivity of rice plants, while *OsNAC3* knock-out decreased the sensitivity of rice shoots but not roots in the presence of ABA.

### *OsNAC3* positively regulates rice tolerance to salt stress

**Fig. 4:**
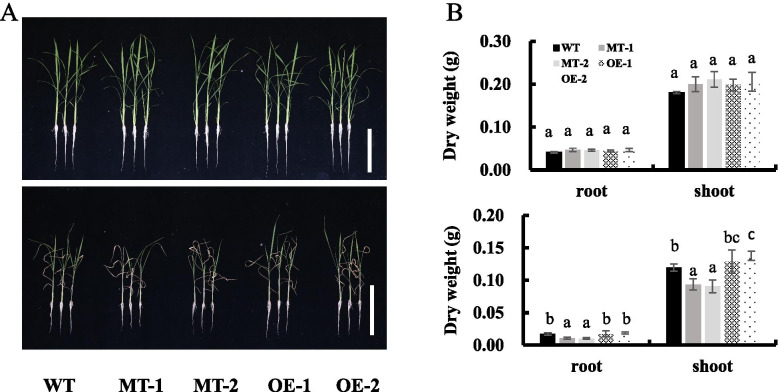
Loss of *OsNAC3* decreases while overexpression increases salt tolerance in rice. (**A**) Growth performance of WT, MT, and OE plants after treatment with 0 (upper panels), 75 (lower panels) mM NaCl for 10 days. Scale bar = 10 cm. (**B**) Dry weight of shoot and root in WT, MT, and OE plants after treatment with 0 (upper panels), 75 (lower panels) mM NaCl. 4-leaf-stage transgenic and Nipponbare rice were treated with 0 or 75 mM NaCl for 10 days. Data represent means ± SD (n = 3). Different letters indicate significant differences (Tukey test, p < 0.05)

To investigate the role of *OsNAC3* in rice salt tolerance, the seedlings of MT, OE, and WT plants were treated with different concentrations of NaCl (0 or 75 mM) for two weeks. We found that in the absence of NaCl, the growth of WT and *OsNAC3*- transgenic plants did not show a significant difference. However, in the presence of 75 mM NaCl, MT lines showed more withered leaves and less dry weight compared with WT lines; on the contrary, OE lines displayed attenuated chlorosis symptoms and greater dry weight (Fig. 4A, B). This phenotypic data suggest that OsNAC3 positively regulates salt tolerance in rice.

### *OsNAC3* knockout increases Na accumulation in shoots of transgenic rice

**Fig. 5:**
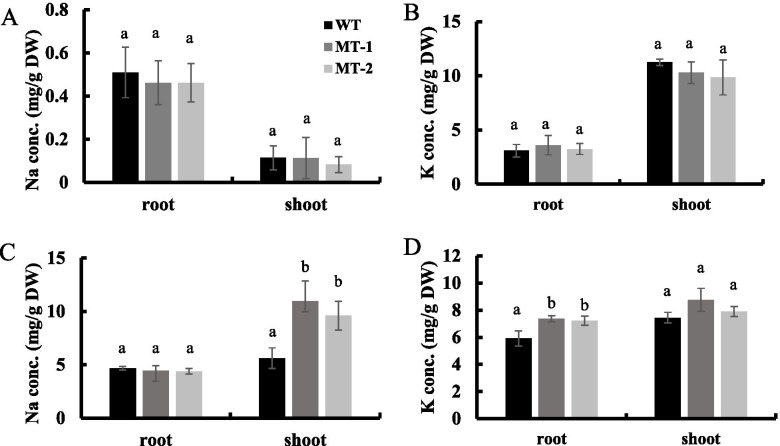
Tissue Na^+^ and K^+^ concentration in WT and *OsNAC3* knockout lines under normal condition or 75 mM NaCl treatment for 10 days. (**A**) Na^+^ concentration in the roots and shoots under normal conditions. (**B**) K^+^ concentration in the roots and shoots under normal conditions. (**C**) Na^+^ concentration in the roots and shoots under 75 mM NaCl treatment. (**D**) K^+^ concentration in the roots and shoots under 75 mM NaCl treatment. 14-day-old transgenic and Nipponbare rice were treated with 0 or 75 mM NaCl for 10 days. Data represent means ± SD (n = 3). Different letters indicate significant differences (Tukey test, p < 0.05)

To test the effect of *OsNAC3* on tissue-specific K^+^/Na^+^ homeostasis, we measured Na^+^ and K^+^ content in WT and *OsNAC3*-knockout plants under control (without NaCl) and salt (75 mM NaCl) conditions. Under control conditions, tissue Na^+^ and K^+^ content showed no differences between the WT and *osnac3* lines (Fig. 5A, B). However, in the presence of 75 mM NaCl, Na^+^ concentration in shoots of two MT lines was about twice (on average) that of WT plants, while no significant difference was found in the roots. Besides, K^**+**^ concentration in roots of two MT lines was only slightly higher than that of the WT plants, while showed no significant difference in the shoots (Fig. 5C, D). These results suggest that OsNAC3 mainly regulates the Na^**+**^ homeostasis of rice shoot under salt stress conditions.

### Knockout of *OsNAC3* affects the expression profiles of several key genes in rice

**Fig. 6:**
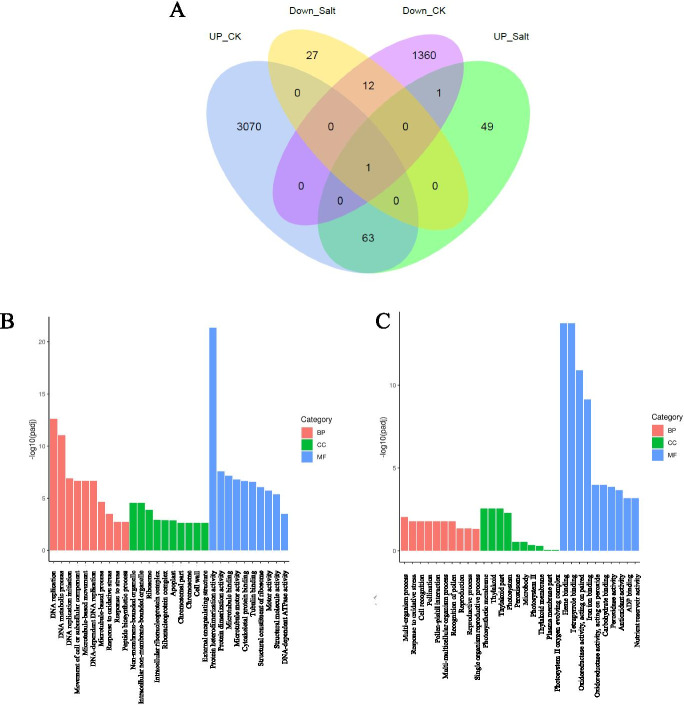
Transcriptome analysis of OsNAC3-regulated genes. (**A**) Venn diagram showing the OsNAC3 regulated genes in MT and WT lines. UP_CK: upregulated genes in MT compared with WT under normal conditions (fold-change > 2). UP_SALT: upregulated genes in MT compared with WT under high salinity (fold-change > 2). DOWN_SALT: the downregulated genes in MT compared with WT under high salinity (fold-change < 0.5). DOWN_CK: the downregulated genes in MT compared with WT under normal conditions (fold-change < 0.5) (**B, C**) GO analysis of downregulated (left) and upregulated (right) genes in MT compared with WT. BP: biological process. CC: cellular component. MF: molecular function. Three biological replicates (n = 3) were performed for each treatment

TFs at large regulate the expression of many genes. To investigate the regulatory function of OsNAC3 in rice, we used high-throughput RNA-seq to analyze the transcriptomes of Nipponbare (WT) and mutant *osnac3* (MT) rice roots under normal and salt conditions. A two-fold change in expression was selected as the threshold to determine the DEGs between the WT and MT lines under normal and salt conditions. We found that there were 3184 up-regulated and 1401 down-regulated DEGs between the WT and MT lines under normal and salt conditions, respectively (Additional file 2). Under the two conditions, 63 and 12 genes were found to be steadily up- and down-regulated, respectively. Therefore, we hypothesized that these genes may be regulated by OsNAC3 (Fig. 6A).

**Fig. 7:**
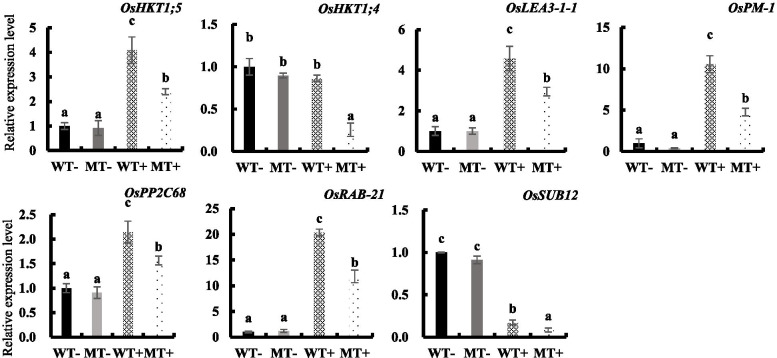
Relative expression levels of potential DEGs under normal and high salinity conditions. WT- and MT- indicate Nipponbare and *OsNAC3*-knockout lines under control conditions. WT+ and MT+ represent Nipponbare and *OsNAC3*-knockout lines treated with 100 mM NaCl for 24 h. 14-day-old *OsNAC3*-knockout and Nipponbare rice were treated with 0 or 100 mM NaCl for 24 h. Data are means ± SD of three biological replicates. Different letters represent significant differences (Tukey test, p < 0.05)

Under normal conditions, the up-regulated genes are mainly involved in DNA replication, DNA metabolic process, and protein heterodimerization activity, while down-regulated genes are mainly related to heme binding, tetrapyrrole binding, and oxidoreductase (Fig. 6 B, C). To verify the RNA-seq data, we performed qRT-PCR to validate the expression level of DEGs and their homologs in rice. As expected, some genes showed different expression levels between WT and MT under two conditions. Under salt conditions, the expression level of seven genes, including *OsRAB21*(Os11g0454300), *OsPP2C68* (Os09g0325700), *OsLEA3–1*(Os05g0542500), *OsPM1* (Os05g0381400), *OsSUB12* (Os02g0198700), *OsHKT1;4* (Os04g0607600) and *OsHKT1;5* (Os01g0307500), was lower in the MT than in WT plants (Fig. 7). These seven genes are considered to be involved in abiotic stress response. Overall, these results indicate that OsNAC3 potentially regulated the stress-related genes in rice to resist salt stress.

## Discussion

In this study, we isolated and functionally characterized a novel NAC transcriptional factor OsNAC3 in rice. OsNAC3 is a nuclear protein with self-activation activities and expresses in root cells. RT-qPCR showed that ABA and NaCl treatment enhanced the root expression of *OsNAC3*. Disruption of *OsNAC3* by CRISPR/Cas9 reduced rice sensitivity to ABA but increased sensitivity to salt stress, while *OsNAC3* overexpression did the opposite. Furthermore, under salt conditions, Na^+^ homeostasis in the shoots and transcript profiles in the roots were altered in the MT plants compared with the WT plants. This indicates a significant role of rice OsNAC3 in ABA response and salt tolerance.

Many rice NAC TFs have been reported to play important roles in ABA response and salt tolerance. For example, ABA and salt stress rapidly induced the expression of *SNAC1* [8]. Overexpression of *SNAC1* significantly enhanced the rice tolerance to drought and salinity through the transcription regulation of many stress-related genes such as *OsSRO1c* and *OsPP18* [8, 25–27]. *OsNAC2* was shown to enhance rice salt tolerance through promoting the expression of ABA biosynthesis genes such as *OsNCED1* and *OsNCED3* [21]. *OsNAC5* and *OsNAC6* were strongly induced by ABA treatment [28–31]. Overexpression of *OsNAC5* and *OsNAC6* improved the tolerance of rice to high salt stress [17, 29, 30, 32]. Phylogenetic analysis showed a close association between OsNAC3 and SNAC1. The amino acid alignment showed that the two proteins shared the conserved domains. Similar to SNAC1, we found that *OsNAC3* expression was also rapidly induced by ABA and salt stress. Notably, *OsNAC3* overexpression improved the salt tolerance of rice. These results suggested that OsNAC3 and SNAC1 may have similar regulatory mechanisms and functional redundancy in ABA response and salt tolerance.

To further investigate the role of OsNAC3 in rice ABA response, phenotypes of MT and OE seedlings were analyzed after exogenous ABA treatment. We found that the shoot and root length of OE plants was significantly suppressed compared with WT; on the contrary, the shoot length of MT plants was significantly more than that of WT plants. These results demonstrated that OsNAC3 plays important role in ABA signal response. In a previous study, we showed that another rice NAC transcription factor OsNAC45 was involved in ABA response. Under exogenous ABA treatment, *OsNAC45*-OE plants displayed a longer length of the shoots and roots, but *OsNAC45*-MT plants showed a shorter length of the shoots and roots compared to the WT plants [33]. Similar phenotypes of *OsNAC3* transgenic plants suggest that it may use the similar ABA response pathway of OsNAC45. Additionally, we found that OsNAC3 and OsNAC45 jointly regulate the expression of *OsPM1* (Plasma Membrane Protein 1) encoding an ABA influx carrier, which is reported to mediate ABA influx functioning in the ABA signaling pathway [34]. This somewhat explains the reasons for similar phenotypes between *OsNAC3* and *OsNAC45* transgenic rice under ABA treatment.

*OsNAC3* knockout changed rice sensitivity to salt stress. Under 75 mM NaCl treatment, MT plants exhibited more wilting leaves compared to WT plants. Moreover, in the absence of NaCl, the roots and shoots concentrations of Na^+^ and K^+^ were similar between the WT and MT plants. In the presence of 75 mM NaCl, shoot Na^+^ concentration significantly increased in MT than in WT, while root K^+^ concentration was only slightly higher in MT. However, under the same treatments, tissue Na^+^ and K^+^ concentrations were similar between WT and *osnac45* lines although *OsNAC45* knockout lines also showed more sensitivity to salt stress than the WT plants. In *osnac45* mutants, more accumulation of reactive oxygen species (ROS) in roots was detected [33]. These differences suggested that *OsNAC3* and *OsNAC45* might be involved in rice salt tolerance through distinct regulatory mechanisms. The RNA-seq and qRT-PCR analysis showed that the expression of some genes encoding Na^+^/K^+^ transporters such as *OsHKT1;4* and *OsHKT1;5* was regulated by OsNAC3. It is well known that HKT (High-affinity K^+^ Transporter) gene family mediate Na^+^ and K^+^ transport in rice [35]. Under salinity stress conditions, OsHKT1;4 and OsHKT1;5 perform Na^+^ unloading in xylem to prevent over accumulation of Na^+^ in shoot [36–38]. Therefore, compared to WT, increased Na^+^ accumulation in MT shoots under salt conditions can be attributed to the lower expression of *OsHKT1;4* and *OsHKT1;5*. These results indicated that *OsNAC3* mutation changes the expression of some Na^+^/K^+^ transporters in roots causing less Na exclusion in roots and more Na root-to-shoot translocation, which in turn increases rice sensitivity to salt stress.

Furthermore, transcriptome sequencing analysis revealed 1401 down-regulated DEGs in the roots after *OsNAC3* knockout under normal and salt conditions. Go enrichment analysis showed that most DEGs were related to heme binding, tetrapyrrole binding, and oxidoreductase. To validate some of the DEGs and their homologs in rice, we performed qRT-PCR and found that *OsLEA3–1, OsRAB-21, OsPM-1, OsPP2C68, OsHKT1;4, OsHKT1;5, OsSUB12* might be regulated by OsNAC3. *OsLEA3–1* is an abiotic stress-induced gene, which encodes for a late embryogenesis abundant (LEA) protein. Overexpression of *OsLEA3–1* significantly increases the rice tolerance to salt and drought stress [39, 40]. Rice RAB (response to abscisic acid) proteins belong to another subgroup of the LEA protein family. In tobacco and rice plants, *OsRAB-21* is upregulated to counter the salinity, drought, and ABA stresses [41, 42]. OsPM1 is an ABA influx carrier. A previous report suggested that *OsPM1* expression is regulated by OsbZIP46, which then plays important role in response to drought stress [34]. Recently, we showed that *OsPM1* expression is regulated by OsNAC45, indicating the regulatory role of OsNAC3 in the ABA signal transduction pathway along with OsbZIP46 and OsNAC45 [33]. OsPP2C68, a member of the PP2C family, is one of the key components of the ABA signal transduction pathway and regulates abiotic stress [43]. OsHKT1;4 and OsHKT1;5 mediating Na^+^ and K^+^ transport are proposed to be major players in root-to-shoot Na^+^ partitioning and K^+^/Na^+^ homeostasis [36–38]. These imply that *OsNAC3* involved in ABA response and salt tolerance might be due to regulate the expression of above-mentioned genes.

Interestingly, OsSUB12 (Submergence Tolerance 12), encoding an ethylene-responsive transcription factor, is homologous to OsSUB1A that mediates rice submergence stress tolerance [44]. Furthermore, analysis of cis-acting elements showed that there are many abiotic stress response-related elements, such as MYB binding sites, W-boxes, and ABRE (ABA-responsive element), in the promoter region of *OsNAC3*. These results suggest that OsNAC3 might play important roles in multiple abiotic stress responses in rice.

## Conclusion

In summary, OsNAC3, a novel nuclear-localized transcription factor, participates in ABA response and salt tolerance through regulating the expression of stress-responsive genes and shoot Na^+^ homeostasis in rice.

## Methods

### Plant materials and growth conditions

We used the wild-type rice (*Oryza sativa* cv Nipponbare), two *OsNAC3*-knockout lines, and two *OsNAC3* overexpression lines in this work. The wild type rice was obtained from rice resources conservation center of Guangxi University. The *OsNAC3* mutants and overexpression lines were constructed in our laboratory (see below). The cultivation of plants conforms to China’s legislation on genetically modified plants. The formal identification of the *OsNAC3*-knockout and overexpression lines was conducted by Xiang Zhang and Yan long. Seeds of the wild-type, *OsNAC3*-knockout and overexpression lines were preserved in our lab, but not in a publicly available herbarium.

Rice seeds were germinated in water at 28 °C for 2 days in darkness. The germinated seeds were then placed on a floating net containing 0.5 mM CaCl_2_ solution at 28 °C and a 12/12 h light/dark cycle. After growing for 4–7 days at 28 °C, plants were cultured in a 4-L plastic spot filled with one-half-strength Kimura B solution (pH 5.6) as described previously [45].

Root, stem, leave, leave sheath, and spike of heading stage rice were harvested for tissue-specific expression analysis of *OsNAC3*. 5-day-old Nipponbare was used to study the induction of *OsNAC3*. For salt stress, rice seedlings were exposed to different concentrations of NaCl (0, 25, 50, 75, 100, and 150 mM) for 12 h or 100 mM NaCl for different times (0, 1, 3, 6, 12, and 24 h). For ABA treatment, rice seedlings were exposed to different concentrations of ABA (0, 6, 12, 30, 50, and 100 μM) for 12 h or 50 μM ABA at different times (0, 1, 3, 6, 12, and 24 h).

### Generation of transgenic rice plants

To create the *OsNAC3*-OE construct, total RNA, extracted from Nipponbare, was reverse transcribed to cDNA by RT-PCR, which was then used as a template to amplify the *OsNAC3* coding region. The full-length cDNA of *OsNAC3* was inserted into the pCAMBIA1300-Ubi vector between the Ubiquitin promoter and nopaline synthase terminator. To create the *OsNAC3*-MT construct, CRISPR/Cas9 gene-editing system was used. The pCRISPR-*OsNAC3* construct with two *OsNAC3*-specific target sites was constructed as reported previously [46]. These constructs were transferred to *Agrobacterium tumefaciens* strain EHA101, which was then transformed into rice cv. Nipponbare. The primers used in this work are listed in Supplementary Table 1.

### Bioinformatics analysis

Amino acid sequences of several stress-response NAC genes were compared with OsNAC3 using MEGA7 with 1000 bootstrap replicates and the neighbor-joining tree (NJT) method. Alignment of OsNAC3, ANAC102, SNAC1, and OsNAC4 proteins was performed with CLUSTAL OMEGA (http://www.clustal.org/omega/) and graphical representation was created with Espript 3.0 (http://espript.ibcp.fr/ESPript/ESPript/).

For promoter analysis, 2000 bp upstream of the transcription start site were retrieved from NCBI (https://www.ncbi.nlm.nih.gov/) and putative cis-elements were searched with Plant CARE database (http://bioinformatics.psb.ugent.be/webtools/plantcare/html/).

### Transactivation activity analysis

For transactivation activity analysis, the coding region of *OsNAC3* was fused in-frame to yeast GAL4 DNA binding domain of vector pGBK-T7, producing the pGBK- *OsNAC3.* pGBK -*OsNAC45* (positive control), pGBK -*OsNAC3*, or pGBK-T7 empty vector (negative control); these vectors were then transformed into yeast strain AH109 according to the Matchmaker Gold Yeast Two-Hybrid System user manual (Clontech). Yeast cells carrying the pGBK- *OsNAC3,* pGBK -*OsNAC45* or empty vector were spotted on SD/Trp- or SD/Trp−/His−/Ade- medium. The plates were photographed after incubation for 3 days at 30 °C.

### Protoplast isolation

The protoplasts were isolated based on the procedures described by Zhang et al. [47] with some modifications. Briefly, seeds of the indica rice 9311 were grew on MS medium in the dark in a growth chamber at 28 °C for two weeks. Leaf sheaths of 50 rice seedlings were harvest and cut into 1 mm pieces using a fresh sharp razor blade. The leaf sheath pieces were quickly transferred into 0.6 M mannitol for a quick plasmolysis treatment, followed by enzymatic digestion in the dark with gentle shaking. After digestion, the protoplasts were collected by filtration through a 50-μm cell strainer. Finally, the protoplasts were resuspended gently in MMG solution (4 mM MES, pH 5.7, 0.6 M mannitol, and 15 mM MgCl_2_). Approximately 1 × 10^6^ cells were used for the following transformation.

### Subcellular localization of OsNAC3

To study the subcellular localization of OsNAC3, the coding region of *OsNAC3* was fused in-frame to GFP of vector pYL322-*GFP*, producing the pYL322-*OsNAC3*-*GFP* construct. pYL322-*OsNAC3*-*GFP,* or vector control; these were co-transformed with nucleus marker (*RFP-OsGhd7*) into rice protoplast as described previously [48]. After 12 h of incubation, fluorescence images were captured by a confocal laser scanning microscope (TCS SP8; Leica).

### Cellular localization of OsNAC3

A 2000 bp region upstream of the transcription start site was PCR amplified from Nipponbare genomic DNA; the coding region of *OsNAC3* without stop codon was PCR amplified from Nipponbare cDNA. Promoter and coding region were cloned into the pCAMBIA1300-*GFP* vector to generate the Pro*OsNAC3-OsNAC3-GFP* construct, which was transfected into *Agrobacterium tumefaciens strain* EHA101 for transformation into rice cv. Nipponbare.

Immunofluorescence assays were performed to detect cellular localization of OsNAC3 as described previously [33]. Briefly, roots of transgenic Nipponbare plants carrying Pro*OsNAC3-OsNAC3-GFP* were embedded in 5% agarose. Next, cross-sections were generated with a micro slicer (VT1000 S, Leica), and the sections were incubated with the rabbit anti-GFP polyclonal antibodies, followed by secondary antibodies (Alexa Fluor 555 goat anti-rabbit IgG; Molecular Probes) at room temperature (RT). Fluorescence images were captured by confocal laser scanning microscope (TCS SP8; Leica); DAPI was used to stain nuclei.

### Seedling growth assay

To examine the growth rate of transgenic rice under ABA treatment, Nipponbare and transgenic rice seeds were germinated on 1/2 MS medium at 28 °C for 2 days in darkness. Then, the seedlings of similar size were transferred to 1/2 MS medium containing 0 or 2 μM ABA in a growth chamber at 28 °C and a 12/12 h light/dark cycle. Shoot and root length were measured after 5 days of incubation.

### Salt stress treatment assay

To investigate the salt tolerance of transgenic rice, 4-leaf-stage transgenic and Nipponbare rice (14-days old) were treated with 0 or 75 mM NaCl for 2 weeks. Then, the roots and shoots were sampled, dehydrated at 70 °C for 3 days, and weighed. The samples were digested with 65% HNO_3_ at 130 °C [49]. The Na^+^ and K^+^ contents in the digested solution were measured by ICP-MS (Plasma Quant MS; Analytik Jena AG).

### RNA-sequencing

Four-leave stage rice (MT and WT) was treated with 0 or 100 mM NaCl for 24 h, and then rice roots were harvested to extract the total RNA for the synthesis of cDNA [50]. RNA-seq was performed on an Illumina Nova Seq platform and the Deseq2 method was used to compare the expression profile of downstream genes in WT and MT. DEGs (differentially expressed genes) were sorted using the criteria |log2_ratio| > 2. DEGs were subjected to GO (Gene Ontology; http://geneontology.org/) analysis and *p*-values were used to assess the significant enrichment of the corresponding category.

## Supplementary Information

**Additional file 1.**

**Additional file 2.**

## Data Availability

All the data supporting the conclusions of this article are provided within the article and in its additional files. All data and materials are available upon reasonable request from the corresponding author.

## Abbreviations

NAC: NAM ATAF and CUC
TFs: transcription factors
RT-PCR: reverse transcription-PCR
qRT-PCR: Quantitative real-time PCR
RFP: red fluorescent protein
RNA-Seq: RNA sequencing
DEGs: Differentially expressed genes
GO: Gene ontology
HKT: High-affinity K^+^ Transporter
