# Supplementary material for: A NAC transcription factor OsNAC3 positively regulates ABA response and salt tolerance in rice
Source: BMC Plant Biol. 2021 Nov 20;21:546. doi: 10.1186/s12870-021-03333-7 (PMC8605558; doi:10.1186/s12870-021-03333-7)
Supplement: Supplementary file 1 — Additional file 1. [file 12870_2021_3333_MOESM1_ESM.docx]

**Additional file 1:**

**
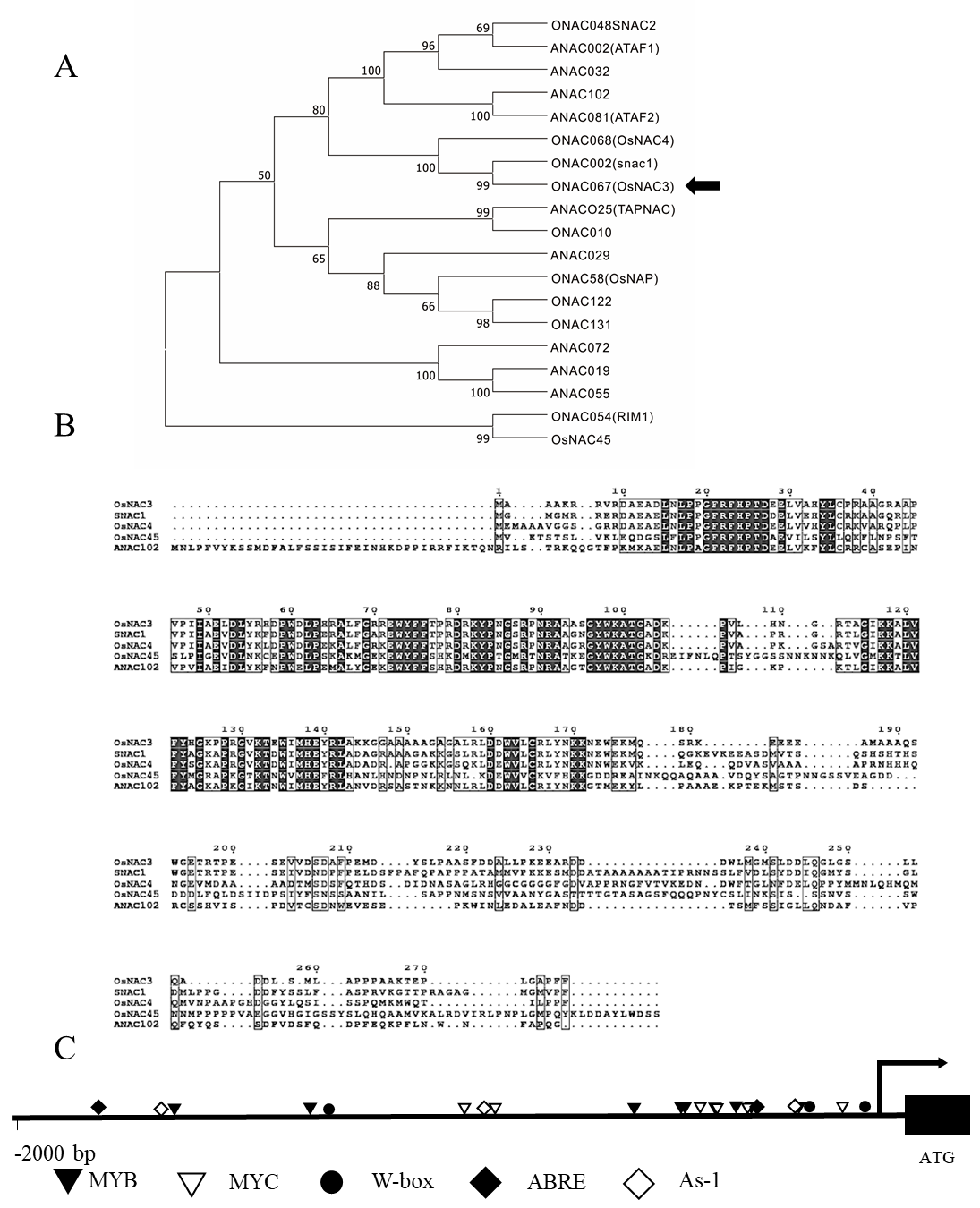
**

**Fig. S1** Bioinformatics analysis of OsNAC3. (**A**) Phylogenetic tree of OsNAC3. 17 homologs sharing with more than 75% identity to OsNAC3, together with OsNAC3 and OsNAC45, were used to construct the phylogenetic tree. Multiple sequence alignment was performed using the Clustal Omega program and the phylogenic tree was created and visualized using Espript3.0. (**B**) Multiple sequence alignment of NAC proteins from rice and Arabidopsis. (**C**) Distribution of cis-acting elements in the promoter region of *OsNAC3*.


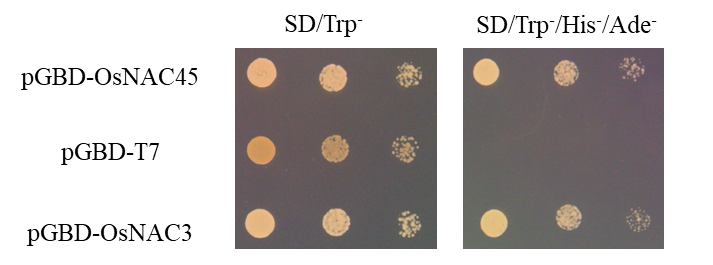


**Fig. S2** Self-activation analysis of OsNAC3. Yeast carrying pGBD-OsNAC45 (positive control), pGBD-T7 (negative control) or pGBD-OsNAC3 were spotted on the SD/Trp- (left) or SD/Trp-/His-/Ade- plates (right) for 3 days at 28 ℃.


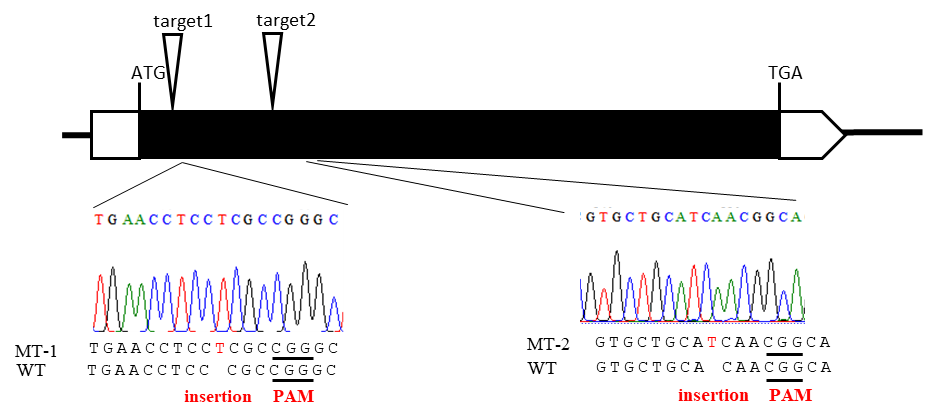


**Fig. S3** *OsNAC3* sequence of two independent mutants generated by CRISPR/Cas9 mutagenesis. The black box indicates the exon.


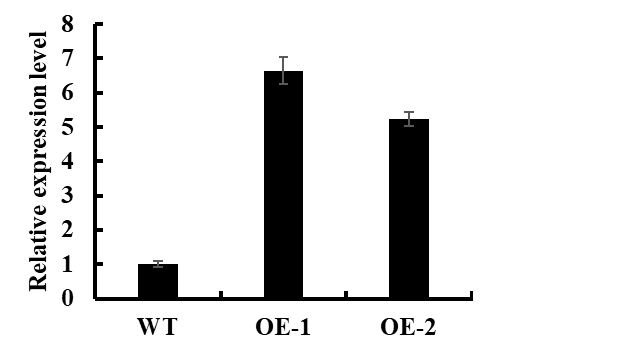


**Fig. S4** Relative expression levels of *OsNAC3* in the *OsNAC3*-overexpression lines (OE-1, OE-2); OE-1 and OE-2 were chosen for further experiments. Data are means ± SD of three biological replicates.

**Table S1. Primers used in this study**

| 225300gRTF | GGCGGACCTGAACCTCCCGCGTTTTAGAGCTAGAAAT |
| --- | --- |
| t225300OsU3T | GCGGGAGGTTCAGGTCCGCCTGCCACGGATCATCTGC |
| OsNAC3-g RT | GACAAGCCCGTGCTGCACAA |
| OsNAC3-u6a | TTGTGCAGCACGGGCTTGTC |
| OsNAC3-1-y-F | CCAACAACCCACCACCCTCA |
| OsNAC3-1-y-R | CGCGTCTCCCCCCACGACTG |
| OsNAC3-2-y-F | GTTTTAGAGCTAGAAAT |
| OsNAC3-2-y-R | CGGCAGCCAAGCCAGCA |
| OsNAC3-pox-F | CCCAAGCTTCATGGCGGCGGCGAAGCGGCGAGT |
| OsNAC3-pox-R | CGCGGATCCTCAGAAGAATGGCGCGCCGA |
| OsNAC3-GFP-F | CCCAAGCTTCATGTTTGACCGTTCGTCTTATTCAAAAG |
| OsNAC3-GFP-R | CGCGGATCCGAAGAATGGCGCGCCGAGCGGC |
| OsNAC3-RT-F | ATCAAGAAGGCGCTCGTGT |
| OsNAC3-RT-R | CGTGCATGATCCACTCCGTC |
| OsRAB21-RT-F | CACACCACAGCAAGAGCTAAGTG |
| OsRAB21-RT-R | TGGTGCTCCATCCTGCTTAAG |
| OsERF3-RT-F | CAGCAATAGCACGGTAGACA |
| OsERF3-RT-R | AGGAGTCGGAGTCACTTTGT |
| OsPP2C68-RT-F | CGCAGCTCCGACAACATCT |
| OsPP2C68-RT-R | GCTGGGTGACACTCTCTCTACAAG |
| OsLEA3-1-RT-F | AATGATTTCCCTTTGGGTC |
| OsLEA3-1-RT-R | CATCAGTACACATCACCCA |
| OsPM1-RT-F | ACACACCGGCCAATCGAT |
| OsPM1-RT-R | AGCGGGAAACACAAAGTGAAG |
